# Short-term calorie restriction enhances adult hippocampal neurogenesis and remote fear memory in a Ghsr-dependent manner

**DOI:** 10.1016/j.psyneuen.2015.09.023

**Published:** 2016-01

**Authors:** Amanda K.E. Hornsby, Yushi T. Redhead, Daniel J. Rees, Michael S.G. Ratcliff, Alex Reichenbach, Timothy Wells, Lewis Francis, Katia Amstalden, Zane B. Andrews, Jeffrey S. Davies

**Affiliations:** a Molecular Neurobiology, Institute of Life Sciences, College of Medicine, Swansea University, UK; b Monash University, Victoria, Australia; c School of Biosciences, Cardiff University, UK; d Texas A&M University, College Station, TX, USA

**Keywords:** Calorie restriction, Ghsr, Ghrelin, Adult hippocampal neurogenesis

## Abstract

The beneficial effects of calorie restriction (CR) have been described at both organismal and cellular levels in multiple organs. However, our understanding of the causal mediators of such hormesis is poorly understood, particularly in the context of higher brain function. Here, we show that the receptor for the orexigenic hormone acyl-ghrelin, the growth hormone secretagogue receptor (Ghsr), is enriched in the neurogenic niche of the hippocampal dentate gyrus (DG). Acute elevation of acyl-ghrelin levels by injection or by overnight CR, increased DG levels of the neurogenic transcription factor, Egr-1. Two weeks of CR increased the subsequent number of mature newborn neurons in the DG of adult wild-type but not Ghsr^−/−^ mice. CR wild-type mice also showed improved remote contextual fear memory. Our findings suggest that Ghsr mediates the beneficial effects of CR on enhancing adult hippocampal neurogenesis and memory.

## Highlights

• The acyl-ghrelin receptor (Ghsr) is expressed in mature granule cell neurons of the dentate gyrus.
• Acyl-ghrelin & calorie restriction increase Egr-1 expression in the dentate gyrus.
• Calorie restriction increases neurogenesis and hippocampal dependent remote contextual fear memory via Ghsr.

## Graphical abstract

**Figure:**
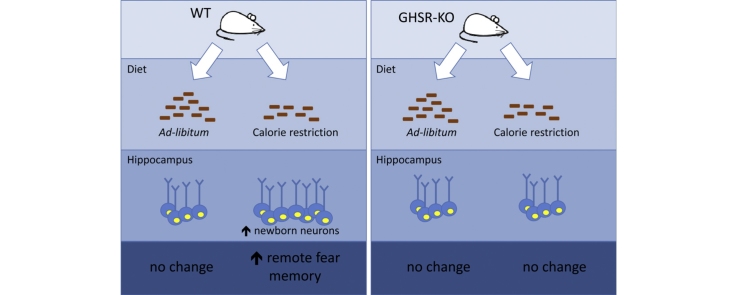

## 1 Introduction

Calorie restriction (CR), in the absence of malnutrition, has beneficial effects on brain function, including reducing the incidence of age-related neurodegenerative disease (Gräff et al., 2013), eliciting anti-depressant behavior (Lutter et al., 2008) and improving memory function in rodents (Fontán-Lozano et al., 2007). In non-human primates, prolonged CR in adulthood decreases the incidence of age-related disease, including measures of brain atrophy (Colman et al., 2009). Whilst in adult humans a 3-month period of CR has been shown to improve memory function (Witte et al., 2009). The physiological mechanism(s) underlying these effects are not fully understood. One process implicated in regulating anxiolytic and mnemonic behavior is adult hippocampal neurogenesis (AHN). This is a form of ongoing plasticity that occurs throughout life involving the birth, differentiation and maturation of new neurons in the adult mammalian dentate gyrus (DG). Decreased neurogenesis has been implicated in the pathogenesis of anxiety and depression (Snyder et al., 2011) as well as cognitive impairment (Yassa et al., 2011) and dementia (Höglinger et al., 2004, Komuro et al., 2015). Recently it has been shown that AHN is essential for distinguishing similar but distinct contexts by laying down non-overlapping memory traces (Clelland et al., 2009, Creer et al., 2010, Nakashiba et al., 2012, Sahay et al., 2011); this form of cognition, termed pattern separation, is impaired in anxiety-disorders (Kheirbek et al., 2012) and cognitive decline (Yassa et al., 2011). Notably, factors such as exercise (Van Praag et al., 2005, Van Praag et al., 1999) and environmental enrichment positively modulate the rate of AHN and performance in pattern separation dependent cognitive tasks (Clelland et al., 2009) and anxiety related tests (Llorens-martín et al., 2010). In addition, reducing the number of calories consumed promotes the survival of newborn cells in the hippocampus (Lee et al., 2002). However, the underlying mediator(s) are not known and it is unclear whether these newborn cells mature into differentiated neurons or if they integrate into hippocampal circuitry to modulate mnemonic processes.

The orexigenic gastrointestinal hormone, acyl-ghrelin, which is elevated during CR (Lutter et al., 2008), is known to cross the BBB and bind to the growth hormone secretagogue receptor (Ghsr) within the hippocampus (Diano et al., 2006). Ghsr is necessary for the anxiolytic effect of CR and exogenous treatment with acyl-ghrelin reduces anxiety behavior (Lutter et al., 2008) and improves performance in spatial learning tasks (Carlini et al., 2010, Diano et al., 2006). Moreover, acyl-ghrelin increases cell proliferation in the hippocampus (Moon et al., 2009) and adult ghrelin deficient mice show reduced rates of new neuron differentiation that were restored to wild-type levels following acyl-ghrelin treatment (Li et al., 2013). These data demonstrate that supra-physiological doses of acyl-ghrelin improve cognition, however, more recently we showed that daily injections of acyl-ghrelin, at a dose similar to plasma concentrations after a 24 h fast, enhanced AHN and pattern separation memory performance (Kent et al., 2015). Based on these data we hypothesize that acyl-ghrelin mediates the neurogenic and cognitive enhancing effects of CR.

Here, we demonstrate that Ghsr is expressed in mature granule cells of the DG and that elevating peripheral acyl-ghrelin, either by injection or CR, increases expression of the zinc finger transcription factor, early growth response 1 (Egr-1) in the DG. Egr-1 is an immediate early gene involved in mitogenesis and differentiation that has recently been implicated in increasing AHN in mice (Veyrac et al., 2013). Using a two-week CR paradigm, paired with a BrdU pulse-chase approach, we demonstrate that CR increases the subsequent generation of adult born mature neurons in a Ghsr-dependent manner. Furthermore, the increase in hippocampal plasticity was accompanied by enhanced remote contextual fear memory, a mnemonic process associated with AHN (Kitamura et al., 2009). Together, these results show that Ghsr is required to mediate the beneficial effects of CR on hippocampal plasticity and memory.

## 2 Materials and methods

### 2.1 Animals

All animal work was carried out with appropriate national and institutional approval at Swansea, Cardiff, Texas A&M and Monash Universities.

#### 2.1.1 Mice

Adult Ghsr-eGFP male mice (generated by the GENSAT project, Rockefeller University) (Spencer et al., 2012) were used (*n *= 4/group). We raised acyl-ghrelin levels *indirectly* via CR (overnight, 16 h fast), directly via injection (1 mg/kg i.p), or with both injection and CR. Acyl-ghrelin injections were performed to coincide with the final hour of the fast before mice were anesthetized with sodium pentobarbital and perfused transcardially with 0.9% NaCl solution, followed by 4% paraformaldehyde (PFA) in 0.1 M phosphate buffer, pH 7.4. Brains were post-fixed in 4% PFA for 24 h and cryoprotected in 30% sucrose.

Adult male and female homozygous loxP TB-flanked Ghsr^−/−^ mice and their wild-type (WT) littermates (a gift from Prof Jeffrey Zigman, UT Southwestern, Texas; (Zigman et al., 2005)) were derived from crosses between animals that were heterozygous for the Ghsr^−/−^ allele and that had been backcrossed >10 generations onto a C57BL6/J genetic background. Ghsr^−/−^ and WT littermate mice (12 weeks old) were individually housed for 7-days under normal laboratory conditions (12 h light: 12 h dark, lights on at 06.00 h) prior to the onset of the study to acclimatize to housing conditions and to assess *ad-libitum* feeding for each genotype and sex. Mice were divided into four groups (*n *= 12/group); *ad-libitum* fed WT, CR WT, *ad-libitum* fed Ghsr^−/−^ and CR Ghsr^−/−^. Each group had 6 male and 6 female mice to allow analysis of sexual dimorphism in the response to CR. CR mice received 70% of the total food consumed by the *ad-libitum* fed group for the first 14-days of the study. To accurately control for CR, food intake from *ad-libitum* fed animals was measured daily; on the subsequent day CR animals would receive 70% of this total. CR feeding was calculated for genotype and sex. On days 4–7 all mice received a daily injection of the thymidine analogue, BrdU (50 mg/kg i.p), to label dividing cells. After 14-days the CR mice were allowed to feed *ad-libitum* for the rest of the study. This experiment was designed to limit acute effects of CR-elevated acyl-ghrelin on LTP and incorporation of GluA1 into excitatory hippocampal synapses (Ribeiro et al., 2014). Furthermore, this BrdU pulse-chase approach was designed to allow specific quantification, via immunohistochemistry, of newborn cells that subsequently mature into neurons. All mice underwent fear memory assessments from day 31 to 45 (see below). Whilst fear conditioning may itself affect ongoing activity-induced neurogenesis in the DG it is unlikely to influence new mature neuron (BrdU^+^/NeuN^+^) number. Mice were killed on day 45 by cervical dislocation under terminal anesthesia, whole brain was removed, immersed in 4% PFA for 24 h at 4 °C, and cryoprotected in 30% sucrose.

### 2.2 Contextual Fear Conditioning (CFC)

CFC was used to assess hippocampus function and memory formation as previously described (Van Woerden et al., 2007), with slight modification. Mice were moved to the test room for 30 min once a day for 6 days prior to conditioning. Equipment was wiped with 70% EtOH before each animal was introduced to the chamber. Mice were pre-exposed to a non-aversive context, a 25 × 25 cm sound-attenuation chamber (Coulbourn Habitest chamber) with a wire grid floor, for 7.5 min. 2 days later each mouse was placed inside a similar but distinct (due to the addition of a colored wall panel) conditioning chamber for 2.5 min before the onset of a 2 s foot shock (0.5 mA). After 2.5 min, a second similar foot shock was delivered, and the mouse was returned to its home cage after another 2.5 min. Mice were tested for context-dependent fear (*i.e* freezing behavior measured in the absence of foot shock) by returning them to the conditioning chamber for 2.5 min 1d, 6d and 12d after conditioning. Presence (1) or absence (0) of freezing behavior was scored every 5 s by a trained observer for 2.5 min (a total of 30 sampling intervals). The observer was blinded to the genotype (the cage cards were replaced by coded cards) but not to feeding regime. Freezing was expressed as a percentage of total number of observations.

### 2.3 Immunohistochemistry

Coronal sections (30 μm) were cut into a 1:12 series along the entire extent of the hippocampus using a freezing-stage microtome (MicroM, ThermoScientific) and collected for IHC. All IHC was performed on free-floating sections at room temperature unless stated otherwise.

For co-localisation of eGFP immunoreactivity sections were washed 3 times in PBS for 5 min, permeabilised in methanol for 2 min at −20 °C, washed again and blocked with 5% normal goat serum (NGS) in PBS plus 0.1% Triton (PBS-T) for 60 min. Sections were incubated overnight at 4 °C in chicken anti-eGFP (1:1000, ab13970, Abcam), washed as before and incubated in goat anti-chicken AF-488 (1:500, Life Technologies, USA) for 30 min in the dark. Sections were washed again prior to a 1 h incubation in either mouse anti-NeuN (1:1000, Millipore, USA), mouse anti-Nestin (1:1000, ab6142, Abcam), rabbit anti-Sox2 (1:500, ab97959, Abcam), rabbit anti-Ki67 (1:500, ab16667, Abcam), rabbit anti-c-Fos (1:500, SC-52, Santa Cruz, USA) or rabbit anti-Egr-1 (1:500, SC-189, Santa Cruz, USA) diluted in PBS-T. Following another wash the sections were incubated with either goat anti-mouse AF-568 or goat anti-rabbit AF-568 (1:500, Life Technologies, USA) for 30 min in the dark. After another wash, including one containing Hoechst stain, sections were mounted onto superfrost+ slides (VWR, France) with prolong-gold anti-fade solution (Life Technologies, USA).

For BrdU/NeuN, sections were treated as described above with the exception that they were first permeabilised in methanol at −20 °C for 2 min and washed prior to pre-treatment with 2N HCl for 30 min at 37 °C followed by washing in 0.1 M borate buffer (pH 8.5) for 10 min. Sections were washed and blocked as above before being incubated overnight at 4 °C in rat anti-BrdU (1:400, AbD Serotec), washed and incubated in goat anti-rat AF-488 (1:500, Life Technologies, USA) for 30 min in the dark. Sections were washed again prior to a 1 h incubation in mouse anti-NeuN (1:1000) diluted in PBS-T. Following another wash the sections were incubated with goat anti-mouse AF-568 (1:500) for 30 min in the dark and mounted as above.

### 2.4 Quantification of labeled cells

A 1:12 series of 30 μm sections (360 μm apart) from each animal was stained and analyzed by fluorescent microscope (Axioscope, Zeiss) or LSM710 META inverted confocal microscope (Zeiss). Immunolabelled cells were manually counted through the *z*-axis using a ×40 objective and throughout the rostro-caudal extent of the granule cell layer. Resulting numbers were divided by the number of coronal sections analyzed and multiplied by the distance between each section to obtain an estimate of the number of cells per DG. For quantification of DG volume, Hoechst nuclear stain was used on tissue sections as above and fluorescent area expressed as μm^2^ per section. For quantification of eGFP with c-Fos or Egr-1 each brain region was anatomically defined using the Mouse Brain Atlas (Paxinos and Franklin, 2012) and cells expressed per mm^2^. Images were processed using Zen (Zeiss) or Image J software. All analyses were performed blind to genotype and treatment.

### 2.5 Statistical analysis

Statistical analyses were carried out using Graphpad Prism 6.0. For comparisons between 2 groups significance was assessed by unpaired Student’s *t*-test. For multiple groups with 1 variable factor a 1-way ANOVA was used, for 2 variable factors a two-way ANOVA was used. Appropriate *post-hoc* tests were used as described. Data are presented as mean ± sem. *, *P *< 0.05; **, *P *< 0.01; ***, *P *< 0.001 were considered significant.

## 3 Results

### 3.1 Ghsr is expressed in mature granule cell neurons of the dentate gyrus

**Fig. 1:**
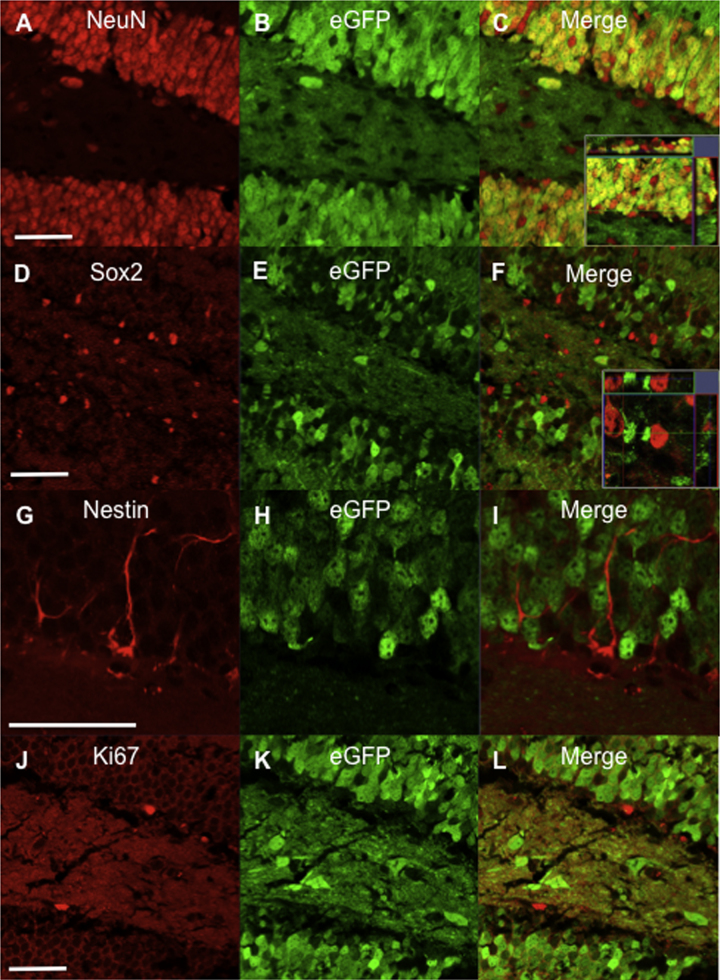
Ghsr is expressed in hippocampal granule cell neurons but not in NSPCs or proliferating cells. Ghsr-eGFP^+^ expression in mature granule cell neurons (NeuN^+^) in the dentate gyrus (**A–C**). No Ghsr-eGFP^+^ co-localisation with Sox2^+^ type II NSC’s (**D**–**F**), nestin^+^ type I NSC’s (**G–I**) or with the cell proliferation marker, Ki67 (**J–L**). *n *= 6 mice per analysis. Scale bar = 50 μm. For interpretation of the references to color in this figure legend, the reader is referred to the web version of the article.

To determine whether the ghrelin receptor, Ghsr, is expressed in higher brain centers associated with regulating AHN we utilized the recently described Ghsr-eGFP reporter mouse (Mani et al., 2014, Reichenbach et al., 2012). As generating antibodies to G-protein coupled receptors with high specificity is difficult due to instability of the purified protein, it has not been possible to accurately probe the cellular phenotype of Ghsr^+^ cells. We overcame this potential constraint by using an antibody raised against eGFP that is fused to the N-terminal of Ghsr. Consistent with previous *in-situ* hybridization results for *Ghsr* mRNA data (Zigman et al., 2006), Ghsr immunoreactivity (Diano et al., 2006) and with a more recent study using the same Ghsr-eGFP mouse model (Mani et al., 2014), Ghsr-eGFP expression was observed extensively throughout the hippocampal DG, including in the sub-granular zone (SGZ) (Fig. 1 and Fig. S1). We further examined the phenotype of eGFP^+^ cells to reveal that Ghsr was extensively co-expressed with the mature neuron marker, NeuN, in the granule cell layer (GCL) of the DG (Fig. 1A–C). We observed eGFP^+^ cells in apposition to both type I (nestin^+^) and type II (Sox2^+^) NSPCs within the SGZ of the DG, however, we found no evidence for Ghsr expression in NSPCs (Fig. 1D–I). In support of this, eGFP^+^ expression was not co-localised with proliferating Ki67^+^ cells within the SGZ (Fig. 1J–L). eGFP immunoreactivity was also observed in hilar interneurons and in dense axon-like projections within the CA3. Very sparse, eGFP immunoreactivity was observed in the CA1 (Fig. S1).

In addition, we observed eGFP immunoreactivity in other extra-hypothalamic brain regions involved in regulating DG neurogenesis. Most notably, in the lateral entorhinal cortex (LEnt) (Stone et al., 2011) and the basolateral amygdala BLA (Kirby et al., 2012) (Fig. S1).

### 3.2 Calorie restriction and acyl-ghrelin induce expression of neurogenic Egr-1 in the dentate gyrus

**Fig. 2:**
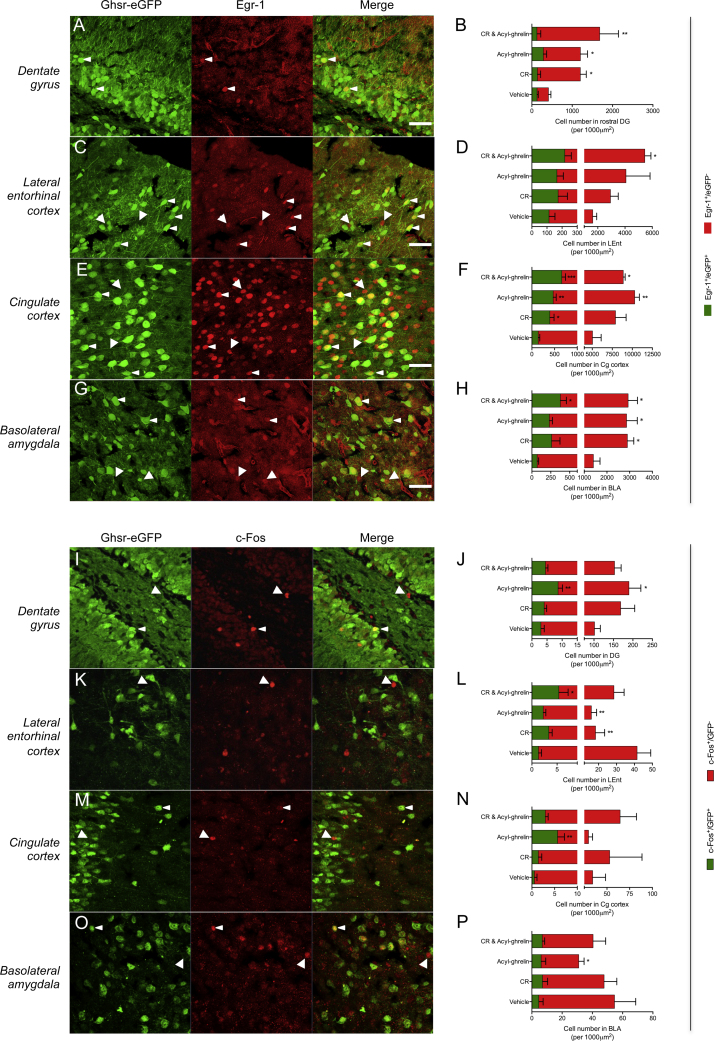
Calorie restriction and acyl-ghrelin increase Egr-1 expression in key learning and memory centers. Representative images of Egr-1^+^ (**A, C, E, G**) and c-Fos^+^ cells (**I, K, M, O**) following CR, acyl-ghrelin or a combination of both CR and acyl-ghrelin. Egr-1^+^ cell number was increased in the DG (**B**), LEnt (**D**), CgC (**F**) and BLA (**H**). c-Fos^+^ cell counts in the DG (**J**), LEnt (**L**), BLA (**P**) and LEnt (**L**). One-way ANOVA with Fishers LSD post-hoc analysis was used for statistical comparison. * *P* < 0.05, ** *P* < 0.01, *** *P* < 0.001 *vs* vehicle treated control. All data shown are mean ± SEM. *n *= 4 mice per group. Scale bar = 40 μm. For interpretation of the references to color in this figure legend, the reader is referred to the web version of the article.

Next, we analyzed whether CR or acyl-ghrelin were able to induce expression of the immediate early gene, Egr-1 and the proto-oncogene, c-Fos, in DG neurons and in brain centers implicated in AHN. We raised acyl-ghrelin levels *directly* via injection, *indirectly* via CR, or with both injection and CR in Ghsr-eGFP mice. 16 h after elevating acyl-ghrelin via CR, expression of Egr-1 was increased in the DG (Fig. 2B, *P *< 0.05) Cingulate Cortex (CgC) Fig. 2F, *P *< 0.05), and BLA (Fig. 2H, *P *< 0.05). Similarly, acyl-ghrelin injection elevated DG (Fig. 2B, *P *< 0.05), CgC (Fig. 2F, *P *< 0.01) and BLA Egr-1 expression (Fig. 2H, *P *< 0.05), whilst the combination of acyl-ghrelin and CR increased Egr-1 expression in DG (Fig. 2B, *P *< 0.01), LEnt (Fig. 2D, *P *< 0.05), CgC (Fig. 2F, *P *< 0.05) and BLA (Fig. 2H, *P *< 0.05). Notably, there was a significant increase in Ghsr-eGFP^+^ cells co-expressing Egr-1 in the CgC (Fig. 2F) in response to CR (*P *< 0.05), acyl-ghrelin (*P *< 0.01) and CR/acyl-ghrelin (*P *< 0.001) treatment. A similar response was also observed in the BLA in response to CR/acyl-ghrelin (Fig. 2H, *P *< 0.05). Conversely, c-Fos expression showed less consistent changes, with a significant decrease observed in the LEnt (Fig. 2L, *P *< 0.01) and BLA (Fig. 2P, *P *< 0.05) after acyl-ghrelin treatment and a similar reduction in the LEnt following CR (Fig. 2L, *P *< 0.01). However, we did observe an increase in c-Fos immunoreactivity in the SGZ of the DG (Fig. 2J) in both the Ghsr-eGFP^−^ (*P *< 0.05) and Ghsr-eGFP^+^ (*P *< 0.01) neurons following treatment with acyl-ghrelin. There was also an increase c-Fos^+^ cell number in the LEnt (Fig. 2L, *P *< 0.05) after CR/acyl-ghrelin and CgC (Fig. 2N, *P *< 0.01) after acyl-ghrelin treatment. A two-way ANOVA revealed that the Ghsr-eGFP^+^ cell population wasn't more sensitive than the Ghsr-eGFP^−^ population to elevated acyl-ghrelin, at least in its Egr-1 and c-Fos immunoreactivity.

**Fig. 3:**
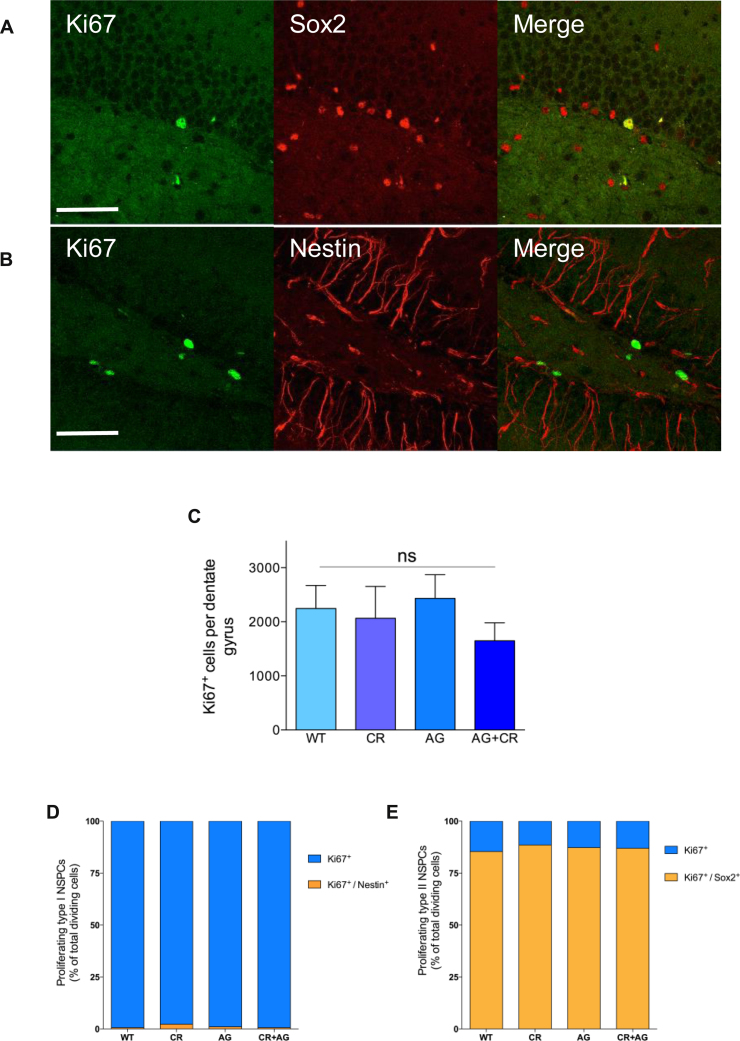
Calorie restriction and acyl-ghrelin do not increase cell proliferation in the DG. Hippocampal cell proliferation (Ki67^+^) (**A**, **B**) was not affected by the acute elevation of acyl-ghrelin, by indirect (CR), direct (acyl-ghrelin injection) or a combination of both means (**C**) (*P* > 0.05). Similarly, treatments did not differentially regulate proliferation rates of either radial type I (Nestin^+^) (**D**) or non-radial type II (Sox2^+^) (**E**) NSPCs (*P* > 0.05). Representative images of Ki67^+^, nestin^+^ and Sox2^+^ cells in the hippocampal DG (**A**, **B**). Data represents mean ± SEM. Scale bar = 50 μm. For interpretation of the references to color in this figure legend, the reader is referred to the web version of the article.

Analysis of cell proliferation revealed that acute elevation of acyl-ghrelin, directly or indirectly, had no effect on DG Ki67^+^ cell number and did not differentially regulate radial type I (Nestin^+^) or non-radial type II (Sox2^+^) NSPCs (Fig. 3).

### 3.3 Calorie restriction increases adult hippocampal neurogenesis and remote contextual fear memory in a Ghsr-dependent manner

**Fig. 4:**
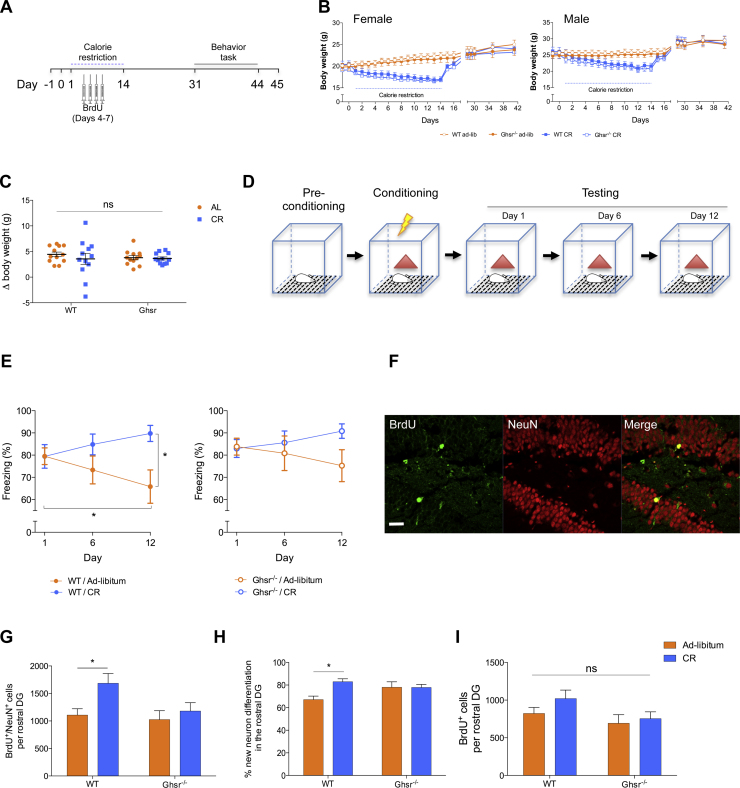
Calorie restriction enhances remote contextual fear memory and adult hippocampal neurogenesis in a Ghsr dependent manner. (**A**) Schematic of experimental paradigm. (**B**) Average daily body weight and (**C**) change in body weight in *ad-libitum* and CR mice over the study. *n *= 12/group. 2-way ANOVA/Tukey’s. (**D**) CFC paradigm. (**E**) Quantification of freezing in absence of foot shock. *n *= 12/group. 2-way RM-ANOVA/Tukey’s: *p < 0.05. (**F**) Representative images of BrdU^+^ (green) and NeuN^+^ (red) labeled new neurons in DG. Scale bar = 50 μm. Quantification of new adult born mature neuron number (**G**), rate of neuronal differentiation in newly divided NSPCs (**H**) and number of new cells (**I**) in the rostral DG. *n *= 12/group. 2-way ANOVA/Tukey’s: *p < 0.05. All data shown are mean ± SEM. For interpretation of the references to color in this figure legend, the reader is referred to the web version of the article.

Finally, as overnight CR increases Egr-1 expression in the DG and DG Egr-1 is associated with promoting the selection and functional integration of newborn cells in the adult DG (Veyrac et al., 2013), we analyzed the impact of a two-week period of CR on the subsequent generation of mature newborn DG neurons in wild-type and Ghsr^−/−^ mice. Both WT and Ghsr^−/−^ mice had similar sex-specific reductions in body weight in response to CR (Fig. 4B), and both genotypes gained weight at a similar rate once they were allowed to feed *ad-libitum* after day 14 (Fig. 4B). Despite 14 days of CR there was no difference in body weight change between the CR and *ad-libitum* fed groups from either genotype over the course of the 45-day period (Fig. 4C, *P *> 0.05).

On day 31, 17 days after the final day of CR we subjected WT and Ghsr^−/−^ mice to hippocampal-dependent contextual fear conditioning (CFC), a paradigm that is sensitive to AHN (Kitamura et al., 2009, Gu et al., 2012). Ghsr^−/−^ and WT littermate mice, which have similar levels of locomotion (Lutter et al., 2008), showed comparable levels of freezing after training in the ‘shock’ context, suggesting that both groups acquired and retained fear memory equally well (2-way ANOVA) reported no effect of genotype (*F* (1,44) = 0.8882, *P *= 0.3511) or treatment (*F* (1,44) = 0.009506, *P *= 0.9228; Fig. 4E). However, over time CR WT mice demonstrated enhanced fear memory. We observed a significant increase in fear memory maintenance 12 days after exposure to the fear condition in CR WT mice compared to *ad-libitum* fed WT littermates. A 2-way repeated measures ANOVA reported a significant interaction between genotype ×  treatment (*F* (6,88) = 2.971, *P* = 0.0109) and a *post-hoc* Tukey multiple comparison reported a significant difference between CR WT and *ad-libitum* fed WT littermates (*P *= 0.0121). No significant differences were reported between Ghsr^−/−^ mice on either diet (*P *= 0.1837; Fig. 4E). Notably, *ad-libitum* fed WT mice displayed progressive extinction of fear memory that reached statistical significance twelve days after conditioning (Day 1 post-conditioning *vs* Day 12 post-conditioning in WT/*ad-libitum* mice, *P *= 0.0179). However, extinction of the fear memory was not observed in *ad-libitum* fed Ghsr^−/−^ mice (Day 1 post-conditioning *vs* Day 12 post-conditioning in Ghsr^−/−^ / *ad-libitum* fed mice, *P *= 0.1889), suggesting that ghrelin receptor signaling may be involved in this process.

Subsequent analysis of brains collected on day 45, 31 days after the last day of CR and 38 days after the last BrdU injection, revealed that CR led to a 52% increase in the number of new adult-born neurons (BrdU^+^/NeuN^+^) in the rostral DG of WT mice compared to *ad-libitum* fed WT mice (2-way ANOVA, main effect of treatment (*F* (1,44) = 5.806, *P *= 0.0202); a *post-hoc* Tukey multiple comparison confirmed a significant difference between CR and *ad-libitum* fed WT mice, *P *= 0.048; Fig. 4G). These data are comparable with our previous findings in rats that a physiological dose of acyl-ghrelin was sufficient to increase AHN in the rostral DG (Kent et al., 2015). In addition, CR increased the proportion of newborn cells that differentiated into mature neurons in WT mice (*P *= 0.0108), but not in Ghsr^−/−^ mice (*P *> 0.99; Fig. 4H). No differences were observed in new cell number with either genotype or treatment (*P *> 0.05; Fig. 4I). Whilst the DG volume in these experimental mice was not quantified, using a separate group of mice we report that WT and Ghsr^−/−^ mice have no overt changes in DG morphology and that genetic ablation of Ghsr does not alter DG area (WT mice, 2095 ± 79.16 μm^2^ *vs* Ghsr^−/−^ mice, 2105 ± 66.46 μm^2^; *n *= 3 per group, *P *= 0.9236). Also, no difference was observed in BrdU^+^/NeuN^+^ cell number in the DG of *ad-libitum* fed wild-type and Ghsr^−/−^ mice (*P *= 0.9812), suggesting that constitutive Ghsr signaling isn't essential for basal AHN. These data show that the CR-mediated enhancement of AHN is dependent on Ghsr.

## 4 Discussion

Our data show that the ghrelin receptor, Ghsr, links energy homeostasis with a form of adult hippocampal plasticity. The presence of Ghsr on mature DG neurons suggest that acyl-ghrelin may modulate NSPCs indirectly, possibly via soluble factors such as BDNF that support AHN (Bekinschtein et al., 2013). Previous work suggests that ghrelin-treatment increases hippocampal BDNF levels in streptozotocin-induced diabetic rats (Ma et al., 2011), however, further studies are required to determine whether this neurotrophic factor is involved in acyl-ghrelin-mediated AHN. Similarly, under stressful conditions, the elevation in circulating acyl-ghrelin (Lutter et al., 2008, Walker et al., 2014) may protect AHN by inhibiting the release of inflammatory cytokines such as interleukin-6 (Beynon et al., 2013), that are known to impair AHN (Monje et al., 2003, Vallières et al., 2002).

Acyl-ghrelin is known to induce c-Fos and Egr-1 expression in mouse hypothalamus (Hewson and Dickson, 2000). We now show that c-Fos^+^ cells are increased in the DG following acyl-ghrelin treatment, but not following acute CR. However, we report for the first time a robust increase in Egr-1^+^ cells in the DG following treatment with either acyl-ghrelin or with CR. The increase in Egr-1 was similarly observed both in DG cells expressing Ghsr and in cells lacking the receptor, suggesting that ghrelin signaling induces network expression of Egr-1 within the DG. Furthermore, Egr-1 immunoreactivity was regulated in other brain regions, including the BLA and the cingulate cortex. Ultimately, a comprehensive dissection of these regions will need to be performed to identify their contribution to CR-mediated AHN and cognition. Nonetheless, our data suggest that Egr-1 may be particularly responsive to adaptations in energetic balance. Notably, hippocampal *Egr-1* expression is rapidly induced by learning and retrieval of memories, its blockade impairs memory formation (Bozon et al., 2003, Jones et al., 2001), particular the re-consolidation of hippocampal dependent contextual fear memories (Lee et al., 2004). More recently, Egr-1 expression in mature DG neurons was shown to be essential for the survival, maturation and integration of newborn adult neurons into the hippocampal circuitry. Furthermore, *Egr-1*-KO mice showed deficits in hippocampal-dependent long-term spatial memory (Veyrac et al., 2013). These findings suggest that the CR-mediated increase in Egr-1 within the DG may support cognition.

Previous studies have demonstrated that CR increases the number of surviving newborn cells, rather than triggering proliferation, in the DG (Lee et al., 2002) in a ghrelin-dependent manner (Kim et al., 2015). However, the impact of CR on new neuron formation in the DG is unknown. These findings prompted us to ask whether a more prolonged period of CR would increase the number of new mature adult born DG neurons. Indeed, we show that a 2-week period of CR, with just a 30% reduction in daily calories, results in a significant increase in new neurons 31 days following the end of the CR period in wild-type but not Ghsr^−/−^ mice, suggesting that ghrelin signaling mediates the neurogenic effect of CR.

Whilst studies using ghrelin reporter mice suggest that the generation of acyl-ghrelin is restricted to the periphery (Sakata et al., 2009), we cannot rule out the possibility that brain-derived ghrelin may influence AHN. In addition, it is possible that currently unknown Ghsr ligands, other than ghrelin, may play a role in promoting AHN in this context. However, as we have previously shown that peripheral treatment with acyl-ghrelin increases AHN (Kent et al., 2015) and that CR is known to elevate plasma ghrelin (Lutter et al., 2008), we suggest that the CR-mediated and Ghsr-dependent increase in AHN is likely induced by acyl-ghrelin.

AHN is necessary for hippocampus-dependent memory and newborn neurons contribute to spatial pattern separation (Clelland et al., 2009). Notably, dendritic synapses of newborn adult neurons show enhanced plasticity between 4 and 6 weeks of age compared with other stages (Ge et al., 2007). At this point they exhibit increased intrinsic excitability, lower activation threshold (Marin-Burgin et al., 2012, Schmidt-Hieber et al., 2004) and recruitment into circuits mediating behavior (Kee et al., 2007, Nakashiba et al., 2012, Tashiro et al., 2007). Optogenetic silencing of 28 day old adult born neurons resulted in impaired retrieval of a contextual fear memory (Gu et al., 2012). To test whether the CR-mediated increase in AHN contributes to hippocampal function our study was designed so that newborn neurons were 4–6 weeks of age during the CFC assessment. In keeping with previous studies we show that the increase in 4–6 week old neurons was associated with enhanced remote contextual fear memory. This improved retrieval of remote memory is consistent with increased re-consolidation over time; a process also associated with AHN (Kitamura et al., 2009, Pan et al., 2012). These data suggest that CR-induced new adult born neurons assume functional roles in hippocampal circuits supporting mnemonic function. However, the extent to which the CR-mediated maintenance of remote fear memory is relevant to the Ghsr-dependent increase in AHN remains to be tested using more specific approaches. In particular, as AHN is essential for accurate pattern separation, testing our experimental paradigm using behavior tests that place a high demand on discrimination is now warranted.

Our findings raise the question, why would CR enhance remote memory? We speculate that in times of hunger the ability to remember an unsafe context would improve the likelihood of re-feeding successfully and thereby increase the chances of survival. This biological trait would confer a selective pressure and may underlie the beneficial effect of CR on longevity observed in a wide range of species.

Ghsr may have therapeutic value in disorders associated with impairments in AHN. As anxiety disorders and enhanced fear responses observed in PTSD are linked with overgeneralization of similar but distinct memories (*i.e* poor discrimination), we suggest that acyl-ghrelin’s anti-anxiety effect (Lutter et al., 2008) may, at least in part, be mediated by promoting AHN. Indeed, this is consistent with recent findings that the action of the P7C3 anti-depressant is dependent upon Ghsr mediated AHN (Walker et al., 2014). In addition, as AHN undergoes age-related decline and is aberrant in pre-clinical models of neurodegenerative diseases such as Alzheimer’s (Komuro et al., 2015) and Parkinson’s disease (Höglinger et al., 2004), activation of the ghrelin/Ghsr axis may be of therapeutic value in alleviating cognitive decline and promoting healthy ageing. Moreover, as diets high in fat reduce neurogenesis (Lindqvist et al., 2006) and impair cognition (Erion et al., 2014), our data suggest that this may be due, at least in part, to the well described reduction in circulating acyl-ghrelin by high fat diet (Tschöp et al., 2001). Interestingly, data has emerged suggesting the presence of a hypothalamic neurogenic zone that may regulate energy balance (for a review see (Rojczyk-Gołȩbiewska et al., 2014)). Given the role of ghrelin in regulating orexigenic neurons in this region (Cowley et al., 2003), studies are warranted to investigate whether the peptide modulates new neuron formation in the hypothalamus.

As the causal factors that mediate exercise- and environmental enrichment-induced AHN have yet to be determined, the identification of acyl-ghrelin/Ghsr as neurogenic modulators represents a significant advance in our understanding of hippocampal plasticity and may provide valuable therapeutic targets.

Together, these findings demonstrate a previously unknown function for CR and Ghsr in enhancing AHN and remote contextual fear memory.

## Conflict of interest

The authors declare no conflict of interest.

## Funding

The funding sources had no role in the conduct of this research.

## Author contributions

AKEH and YR performed experiments and contributed to analyzing the data. DR, MR and YR assisted in histology. AR, TW, LF and KA performed experiments. ZBA assisted in experiment design and manuscript preparation. JSD designed and performed the experiments, analyzed the data and wrote the manuscript.

## References

[bib0005] Bekinschtein P., Kent B.A., Oomen C.A., Clemenson G.D., Gage F.H., Saksida L.M., Bussey T.J. (2013). BDNF in the dentate gyrus is required for consolidation of pattern-separated memories. Cell Rep..

[bib0010] Beynon A.L., Brown M.R., Wright R., Rees M.I., Sheldon I.M., Davies J.S. (2013). Ghrelin inhibits LPS-induced release of IL-6 from mouse dopaminergic neurones. J. Neuroinflammation.

[bib0015] Bozon B., Bozon B., Davis S., Davis S., Laroche S., Laroche S. (2003). A requirement for the immediate early gene *zif268* in reconsolidation of recognition memory after retrievale. Neuron.

[bib0020] Carlini V.P., Perez M.F., Salde E., Schiöth H.B., Ramirez O.A., de Barioglio S.R. (2010). Ghrelin induced memory facilitation implicates nitric oxide synthase activation and decrease in the threshold to promote LTP in hippocampal dentate gyrus. Physiol. Behav..

[bib0025] Clelland C.D., Choi M., Romberg C., Clemenson G.D., Fragniere a., Tyers P., Jessberger S., Saksida L.M., Barker R.a., Gage F.H., Bussey T.J. (2009). A functional role for adult hippocampal neurogenesis in spatial pattern separation. Science.

[bib0030] Colman R.J., Anderson R.M., Johnson S.C., Kastman E.K., Simmons H.a., Kemnitz J.W., Weindruch R. (2009). Caloric restriction delays disease onset and mortality in rhesus monkeys. Science.

[bib0035] Cowley M.a, Smith R.G., Diano S., Tschöp M., Pronchuk N., Grove K.L., Strasburger C.J., Bidlingmaier M., Esterman M., Heiman M.L., Garcia-Segura L.M., Nillni E.a, Mendez P., Low M.J., Sotonyi P., Friedman J.M., Liu H., Pinto S., Colmers W.F., Cone R.D., Horvath T.L. (2003). The distribution and mechanism of action of ghrelin in the CNS demonstrates a novel hypothalamic circuit regulating energy homeostasis. Neuron.

[bib0040] Creer D.J., Romberg C., Saksida L.M., van Praag H., Bussey T.J. (2010). Running enhances spatial pattern separation in mice. Proc. Natl. Acad. Sci. U. S. A..

[bib0045] Diano S., Farr S.A., Benoit S.C., McNay E.C., da Silva I., Horvath B., Gaskin F.S., Nonaka N., Jaeger L.B., Banks W.A., Morley J.E., Pinto S., Sherwin R.S., Xu L., Yamada K.a., Sleeman M.W., Tschöp M.H., Horvath T.L. (2006). Ghrelin controls hippocampal spine synapse density and memory performance. Nat. Neurosci..

[bib0050] Erion J.R., Wosiski-Kuhn M., Dey A., Hao S., Davis C.L., Pollock N.K., Stranahan A.M. (2014). Obesity elicits interleukin 1-mediated deficits in hippocampal synaptic plasticity. J. Neurosci..

[bib0055] Fontán-Lozano A., Sáez-Cassanelli J.L., Inda M.C., de los Santos-Arteaga M., Sierra-Domínguez S.A., López-Lluch G., Delgado-García J.M., Carrión A.M. (2007). Caloric restriction increases learning consolidation and facilitates synaptic plasticity through mechanisms dependent on NR2B subunits of the NMDA receptor. J. Neurosci..

[bib0060] Ge S., Yang C.H., Hsu K., Sen Ming G.L., Song H. (2007). A critical period for enhanced synaptic plasticity in newly generated neurons of the adult brain. Neuron.

[bib0065] Gräff J., Kahn M., Samiei A., Gao J., Ota K.T., Rei D., Tsai L.-H. (2013). A dietary regimen of caloric restriction or pharmacological activation of SIRT1 to delay the onset of neurodegeneration. J. Neurosci..

[bib0070] Gu Y., Arruda-Carvalho M., Wang J., Janoschka S.R., Josselyn S.a, Frankland P.W., Ge S. (2012). Optical controlling reveals time-dependent roles for adult-born dentate granule cells. Nat. Neurosci..

[bib0075] Hewson A.K., Dickson S.L. (2000). Systemic administration of ghrelin induces fos and Egr-1 proteins in the hypothalamic arcuate nucleus of fasted and fed rats. J. Neuroendocrinol..

[bib0080] Höglinger G.U., Rizk P., Muriel M.P., Duyckaerts C., Oertel W.H., Caille I., Hirsch E.C. (2004). Dopamine depletion impairs precursor cell proliferation in Parkinson disease. Nat. Neurosci..

[bib0085] Jones M.W., Errington M.L., French P.J., Fine A., Bliss T.V.P., Garel S., Charnay P., Bozon B., Laroche S., Davis S. (2001). A requirement for the immediate early gene Zif268 in the expression of late LTP and long-term memories. Nat. Neurosci..

[bib0090] Kee N., Teixeira C.M., Wang A.H., Frankland P.W. (2007). Preferential incorporation of adult-generated granule cells into spatial memory networks in the dentate gyrus. Nat. Neurosci..

[bib0095] Kent B.A., Beynon A.L., Hornsby A.K.E., Bekinschtein P., Bussey T.J., Davies J.S., Saksida L.M. (2015). The orexigenic hormone acyl-ghrelin increases adult hippocampal neurogenesis and enhances pattern separation. Psychoneuroendocrinology.

[bib0100] Kheirbek M.A., Klemenhagen K.C., Sahay A., Hen R. (2012). Neurogenesis and generalization: a new approach to stratify and treat anxiety disorders. Nat. Neurosci..

[bib0105] Kim Y., Kim S., Kim C., Sato T., Kojima M., Park S. (2015). Ghrelin is required for dietary restriction-induced enhancement of hippocampal neurogenesis: lessons from ghrelin knockout mice. Endocr. J..

[bib0110] Kirby E.D., Friedman A.R., Covarrubias D., Ying C., Sun W.G., Goosens K.A., Sapolsky R.M., Kaufer D. (2012). Basolateral amygdala regulation of adult hippocampal neurogenesis and fear-related activation of newborn neurons. Mol. Psychiatry.

[bib0115] Kitamura T., Saitoh Y., Takashima N., Murayama A., Niibori Y., Ageta H., Sekiguchi M., Sugiyama H., Inokuchi K. (2009). Adult neurogenesis modulates the hippocampus-dependent period of associative fear memory. Cell.

[bib0120] Komuro Y., Xu G., Bhaskar K., Lamb B.T. (2015). Human tau expression reduces adult neurogenesis in a mouse model of tauopathy. Neurobiol. Aging.

[bib0125] Lee J., Seroogy K.B., Mattson M.P. (2002). Dietary restriction enhances neurotrophin expression and neurogenesis in the hippocampus of adult mice. J. Neurochem..

[bib0130] Lee J.L.C., Everitt B.J., Thomas K.L. (2004). Independent cellular processes for hippocampal memory consolidation and reconsolidation. Science.

[bib0135] Li E., Chung H., Kim Y., Kim D., Ryu J. (2013). Ghrelin directly stimulates adult hippocampal neurogenesis: implications for learning and memory. Endocrine J..

[bib0140] Lindqvist A., Mohapel P., Bouter B., Frielingsdorf H., Pizzo D., Brundin P., Erlanson-Albertsson C. (2006). High-fat diet impairs hippocampal neurogenesis in male rats. Eur. J. Neurol..

[bib0145] Llorens-martín M., Torres-alemán I., Trejo J.L. (2010). Exercise modulates insulin-like growth factor 1-dependent and -independent effects on adult hippocampal neurogenesis and behaviour. Mol. Cell. Neurosci..

[bib0150] Lutter M., Sakata I., Osborne-lawrence S., Rovinsky S.A., Anderson J.G., Jung S., Birnbaum S., Yanagisawa M., Elmquist J.K., Nestler E.J., Zigman J.M. (2008). The orexigenic hormone ghrelin defends against depressive symptoms of chronic stress. Nat. Neurosci..

[bib0155] Ma L.-Y., Zhang D.-M., Tang Y., Lu Y., Zhang Y., Gao Y., Xia L., Zhao K.-X., Chai L.-Y., Xiao Q. (2011). Ghrelin-attenuated cognitive dysfunction in streptozotocin-induced diabetic rats. Alzheimer Dis. Assoc. Disord..

[bib0160] Mani B.K., Walker A.K., Lopez Soto E.J., Raingo J., Lee C.E., Perelló M., Andrews Z.B., Zigman J.M. (2014). Neuroanatomical characterization of a growth hormone secretagogue receptor-green fluorescent protein reporter mouse. J. Comp. Neurol..

[bib0165] Marin-Burgin A., Mongiat L.A., Pardi M.B., Schinder A.F. (2012). Unique processing during a period of high excitation/inhibition balance in adult-born neurons. Science.

[bib0170] Monje M.L., Toda H., Palmer T.D. (2003). Inflammatory blockade restores adult hippocampal neurogenesis. Science.

[bib0175] Moon M., Hwang L., Park S. (2009). Ghrelin regulates hippocampal neurogenesis in adult mice. Endocr. J..

[bib0180] Nakashiba T., Cushman J.D., Pelkey K.A., Renaudineau S., Buhl D.L., McHugh T.J., Barrera V.R., Chittajallu R., Iwamoto K.S., McBain C.J., Fanselow M.S., Tonegawa S. (2012). Young dentate granule cells mediate pattern separation, whereas old granule cells facilitate pattern completion. Cell.

[bib0185] Pan Y.-W., Chan G.C.K., Kuo C.T., Storm D.R., Xia Z. (2012). Inhibition of adult neurogenesis by inducible and targeted deletion of ERK5 mitogen-activated protein kinase specifically in adult neurogenic regions impairs contextual fear extinction and remote fear memory. J. Neurosci..

[bib0190] Paxinos, G., Franklin, K., 2012. Paxinos and Franklin's the Mouse Brain in Stereotaxic Coordinates 4th Edition.

[bib0195] Reichenbach A., Steyn F.J., Sleeman M.W., Andrews Z.B. (2012). Ghrelin receptor expression and colocalization with anterior pituitary hormones using a GHSR-GFP mouse line. Endocrinology.

[bib0200] Ribeiro L.F., Catarino T., Santos S.D., Benoist M., van Leeuwen J.F., Esteban J.A., Carvalho A.L. (2014). Ghrelin triggers the synaptic incorporation of AMPA receptors in the hippocampus. Proc. Natl. Acad. Sci. U. S. A..

[bib0205] Rojczyk-Gołȩbiewska E., Pałasz A., Wiaderkiewicz R. (2014). Hypothalamic subependymal niche: a novel site of the adult neurogenesis. Cell. Mol. Neurobiol..

[bib0210] Sahay A., Scobie K.N., Hill A.S., O'Carroll C.M., Kheirbek M.a., Burghardt N.S., Fenton A.A., Dranovsky A., Hen R. (2011). Increasing adult hippocampal neurogenesis is sufficient to improve pattern separation. Nature.

[bib0215] Sakata I., Nakano Y., Osborne-lawrence S., Rovinsky S.A., Lee C.E., Perello M., Anderson J.G., Coppari R., Xiao G., Lowell B.B., Elmquist J.K., Zigman J.M. (2009). Characterization of a novel ghrelin cell reporter mouse. Regul. Pept..

[bib0220] Schmidt-Hieber C., Jonas P., Bischofberger J. (2004). Enhanced synaptic plasticity in newly generated granule cells of the adult hippocampus. Nature.

[bib0225] Snyder J.S., Soumier A., Brewer M., Pickel J., Cameron H.A. (2011). Adult hippocampal neurogenesis buffers stress responses and depressive behaviour. Nature.

[bib0230] Spencer S.J., Xu L., Clarke M.A., Lemus M., Reichenbach A., Geenen B., Kozicz T., Andrews Z.B. (2012). Ghrelin regulates the hypothalamic-pituitary-adrenal axis and restricts anxiety after acute stress. Biol. Psychiatry.

[bib0235] Stone S.S.D., Teixeira C.M., Devito L.M., Zaslavsky K., Josselyn S.A., Lozano A.M., Frankland P.W. (2011). Stimulation of entorhinal cortex promotes adult neurogenesis and facilitates spatial memory. J. Neurosci..

[bib0240] Tashiro A., Makino H., Gage F.H. (2007). Experience-specific functional modification of the dentate gyrus through adult neurogenesis: a critical period during an immature stage. J. Neurosci..

[bib0245] Tschöp M., Weyer C., Tataranni P.A., Devanarayan V., Ravussin E., Heiman M.L. (2001). Circulating ghrelin levels are decreased in human obesity. Diabetes.

[bib0250] Vallières L., Campbell I.L., Gage F.H., Sawchenko P.E. (2002). Reduced hippocampal neurogenesis in adult transgenic mice with chronic astrocytic production of interleukin-6. J. Neurosci..

[bib0255] Van Praag H., Kempermann G., Gage F.H. (1999). Running increases cell proliferation and neurogenesis in the adult mouse dentate gyrus. Nat. Neurosci..

[bib0260] Van Praag H., Shubert T., Zhao C., Gage F.H. (2005). Exercise enhances learning and hippocampal neurogenesis in aged mice. J. Neurosci..

[bib0265] Van Woerden G.M., Harris K.D., Hojjati M.R., Gustin R.M., Qiu S., de Avila Freire R., Jiang Y., Elgersma Y., Weeber E.J. (2007). Rescue of neurological deficits in a mouse model for Angelman syndrome by reduction of alphaCaMKII inhibitory phosphorylation. Nat. Neurosci..

[bib0270] Veyrac A., Gros A., Bruel-jungerman E., Rochefort C., Kleine Borgmann F.B., Jessberger S., Laroche S. (2013). Zif268/egr1 gene controls the selection, maturation and functional integration of adult hippocampal newborn neurons by learning. Proc. Natl. Acad. Sci..

[bib0275] Walker a.K., Rivera P.D., Wang Q., Chuang J.-C., Tran S., Osborne-Lawrence S., Estill S.J., Starwalt R., Huntington P., Morlock L., Naidoo J., Williams N.S., Ready J.M., Eisch a.J., Pieper a.a., Zigman J.M. (2014). The P7C3 class of neuroprotective compounds exerts antidepressant efficacy in mice by increasing hippocampal neurogenesis. Mol. Psychiatry.

[bib0280] Witte A.V., Fobker M., Gellner R., Knecht S., Floel A. (2009). Caloric restriction improves memory in elderly humans. Proc. Natl. Acad. Sci..

[bib0285] Yassa M.a., Mattfeld A.T., Stark S.M., Stark C.E.L. (2011). Age-related memory deficits linked to circuit-specific disruptions in the hippocampus. Proc. Natl. Acad. Sci. U. S. A..

[bib0290] Zigman J.M., Jones J.E., Lee C.E., Saper C.B., Elmquist J.K. (2006). Expression of ghrelin receptor mRNA in the rat and the mouse brain. J. Comp. Neurol..

[bib0295] Zigman J.M., Nakano Y., Coppari R., Balthasar N., Marcus J.N., Lee C.E., Jones J.E., Deysher A.E., Waxman A.R., White R.D., Williams T.D., Lachey J.L., Seeley R.J., Lowell B.B., Elmquist J.K. (2005). Mice lacking ghrelin receptors resist the development of diet-induced obesity. J. Clin. Invest..

