# Supplementary material for: Short-term calorie restriction enhances adult hippocampal neurogenesis and remote fear memory in a Ghsr-dependent manner
Source: Psychoneuroendocrinology. 2016 Jan;63:198–207. doi: 10.1016/j.psyneuen.2015.09.023 (PMC4686051; doi:10.1016/j.psyneuen.2015.09.023)
Supplement: Supplementary file 1 [file mmc1.pdf]

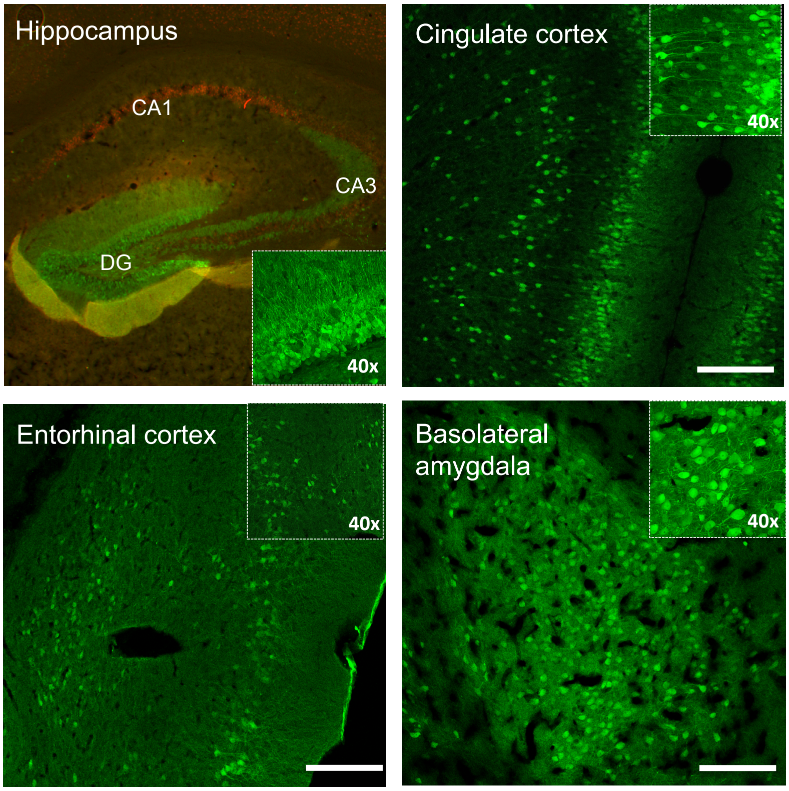

Figure S1. Ghsr is expressed in learning and memory related centres in the adult brain. Adult male Ghsr-eGFP mice confirm expression of Ghsr in extra-hypothalamic sites, including the hippocampus, entorhinal cortex, cingulate cortex and basolateral amygdala. Scale bar = 50μm.
